# Contribution of Shockwave Therapy in the Functional Rehabilitation Program of Patients with Patellofemoral Pain Syndrome

**DOI:** 10.3390/jcm13237260

**Published:** 2024-11-29

**Authors:** Marius Neculăeș, Pablo Hernandez-Lucas, Ioja Ioana-Bianca, Paul Lucaci

**Affiliations:** 1 Faculty of Physical Education and Sport, “Alexandru Ioan Cuza” University of Iași, 3 Toma Cozma Street, 700554 Iasi, Romania; marius.neculaes@uaic.ro (M.N.); bioana0311@yahoo.com (I.I.-B.); paul.lucaci@uaic.ro (P.L.); 2 Faculty of Physiotherapy, University of Vigo, Campus a Xunqueira, s/n., 36005 Pontevedra, Spain

**Keywords:** physiotherapy, pain relief, knee pain, recovery

## Abstract

**Background**: Patellofemoral pain syndrome is a condition with an increasing incidence in recent years, being known as the most common cause of knee pain in adults and adolescents. Undiagnosed and untreated, this condition can worsen over time. The aggravation leads to an increase in the intensity of the pain and the risk of injury, along with an increase in stress on the other joints of the lower limb. The objective of this study was to evaluate the contribution of shockwave therapy to a functional rehabilitation programme for patients with patellofemoral pain syndrome. **Materials and Methods:** The study was carried out on a group of 64 subjects (32 males and 32 females), aged between 20 and 39 years. The subjects were divided into two groups: 32 subjects who followed a program of functional rehabilitation based on low- and medium-frequency electrotherapy, ultrasound and laser therapy, along with a physical therapy program lasting approximately 3 weeks, and 32 subjects who followed a functional rehabilitation program based on shockwave therapy and specific physical therapy exercises lasting approximately 3 weeks. **Results:** Following the protocols applied to the two groups, the pain reported by the patients decreased, while the functional parameters of the knee improved, better results being obtained in the group that performed shock wave therapy together with specific physical therapy programs (Cohen Index 5916, *p* < 0.001). **Conclusions:** This study indicates that radial shockwave therapy combined with physiotherapy may provide additional benefits for patellofemoral syndrome, including greater pain reduction and improved joint mobility, compared to traditional treatments. However, further research is needed to confirm these findings and their broader clinical applicability.

## 1. Introduction

Patellofemoral pain syndrome, also known as patellofemoral syndrome, is characterised by pain in the anterior region of the knee, particularly around the patella, and typically develops progressively [1]. Although the exact cause of patellofemoral syndrome remains unclear, it is widely considered to be multifactorial. This condition primarily affects young women and is often linked to biomechanical imbalances, quadriceps weakness, hip external rotator deficits, and a sedentary lifestyle [2,3,4,5,6,7,8,9,10,11,12]. Moreover, the psychological aspect plays a crucial role in the development of patellofemoral syndrome, which should not be underestimated [13,14,15,16].

Currently, the diagnosis of this condition is based on a detailed assessment of the patient’s medical history, complemented by diagnostic tests such as magnetic resonance imaging and ultrasound [17].

The rehabilitation of patients with patellofemoral syndrome requires an integrated and multidisciplinary approach [18,19,20,21]. Thanks to the wide availability of physical modalities, in recent years, the management of this condition has evolved to include numerous combined therapy approaches incorporating therapeutic exercise interventions alongside traditional biophysical agents such as cryotherapy, ultrasound, and transcutaneous electrical nerve stimulation, as well as taping [22,23,24,25,26]. Furthermore, the use of shockwave therapy has recently emerged as a novel and promising intervention in the rehabilitation of patellofemoral syndrome, as it has been shown to promote tissue healing and reduce pain in various musculoskeletal conditions [27,28]. There are two main types of extracorporeal shockwave therapy (ESWT): focused (F-ESWT) and radial pressure waves (R-PWs). F-ESWT generates concentrated acoustic waves that penetrate deeply into tissues, making it ideal for localised injuries such as chronic tendinopathies. In contrast, R-PWs produce dispersed waves with lower intensity and depth, better suited for treating superficial or broader areas, such as muscles and soft tissues. Both modalities offer therapeutic benefits, including tissue regeneration stimulation and pain reduction, with the choice depending on the pathology and treatment objectives [29]. These findings highlight the potential of shockwave therapy as a valuable option in the management of patellofemoral syndrome.

Given its potential to improve clinical outcomes, this pilot study aims to evaluate the effectiveness of shockwave therapy combined with physiotherapy programmes in patients with patellofemoral syndrome. It is hypothesised that this therapeutic combination will result in a significant reduction in pain and superior functional improvement compared to low-frequency electrotherapy, laser, ultrasound, and other physiotherapy protocols. 

## 2. Materials and Methods

This pilot quasi-experimental study aimed to assess the efficacy of two rehabilitation protocols in patients with patellofemoral syndrome. The sample was randomly divided into two groups, allowing for a comparison of results between them. Participants were voluntarily recruited and provided informed consent prior to the start of the study.

The study was conducted in accordance with the principles of the Declaration of Helsinki and was approved by the Ethics Committee of the Faculty of Physical Education and Sport at “Alexandru Ioan Cuza” University of Iași (approval code: 5/2024, 28 March 2024).

Both groups received treatment under the supervision of experienced physiotherapists, and data were collected before and after the intervention to evaluate the effects. The TiDIER-T recommendations were followed to accurately document the procedures, ensuring the study’s replication and transparency.

### 2.1. Study Participants

A total of 64 subjects, aged between 20 and 39 years and diagnosed with patellofemoral syndrome, were included in the study. Patients were selected from the Kinego clinic in Iași, Romania, where the necessary diagnostic and therapeutic equipment was available, including electrotherapy devices and essential physiotherapy tools for treating the patients.

The subjects included in the study reported pain in the anterior region of the knee, with no history of trauma or signs of significant degenerative changes in the femoropatellar joint, as determined by radiographic evaluation. The diagnosis was confirmed by a specialist physician using musculoskeletal ultrasound or magnetic resonance imaging, which revealed specific signs of inflammation in the patellar and quadriceps tendons.

The initial and final assessments were conducted by the same team of therapists, each with over 15 years of rehabilitation experience, ensuring consistency in data collection. During clinic visits, patients were evaluated individually, and rehabilitation protocols were administered by physiotherapists with at least 10 years of clinical experience, guaranteeing personalised attention and the precise execution of recovery exercises. The initial assessment took place at the beginning of the treatment sessions, while the final evaluation was performed one week after completing the functional rehabilitation programmes. This timing ensured the objectivity of the results and confirmed their sustainability over time.

Outcome measures included pain intensity, assessed using the Visual Analogue Scale (VAS); joint mobility, measured with a goniometer to determine the active range of motion; and the suprapatellar perimeter, measured with a tape placed 10 centimetres above the patella. These metrics provided a comprehensive evaluation of the patients’ progress and an in-depth assessment of the intervention’s effectiveness.

### 2.2. Study Groups and Protocols

The subjects were randomly assigned in a 1:1 ratio to two intervention groups using a computer-generated sequence in Microsoft Excel (v 17.0), ensuring an even and unbiased distribution. This randomisation process was concealed from participants, helping to minimise bias and enhance the internal validity of the study.

Group 1 (*n* = 32): This group followed a functional rehabilitation programme (Supplementary Material S1) and additionally undertook low-frequency electrotherapy with TENS, medium-frequency interferential current, ultrasound, and laser therapy, along with a structured physical therapy programme lasting approximately three weeks.

Group 2 (*n* = 32): This group received a functional rehabilitation programme (Supplementary Material S1) and additionally undertook shockwave therapy in combination with a specific physical therapy regimen for the same duration.

### 2.3. Intervention Details

For Protocol 1 (Group 1), TENS therapy was applied for 15 min with a frequency of 0–100 Hz, using two electrodes: one placed on the patellar tendon and the other on the suprapatellar area. Interferential current was administered for 10 min at the same frequency, with electrodes positioned medial and lateral to the knee joint. Ultrasound therapy was performed on the affected knee for 6 min at an intensity of 1.2 W/cm^2^, using circular movements around the patella without direct contact. Laser therapy was delivered in two phases to target tendinitis, with Phase A using 200 J/cm^2^ at a duty cycle of 100%, and Phase B using 10 J/cm^2^ at a duty cycle of 80%, for a total duration of 8 min. All these procedures were carried out using the BTL-5825SL COMBI device (BTL Industries, Prague, Czech Republic), with the patients positioned supine and the knee in mild flexion, supported by a towel under the popliteal area.

The physical therapy programme was carried out three times per week, with each session lasting 50 min. The exercises aimed to reduce localised knee pain, maintain or restore knee mobility, prevent quadriceps atrophy, and re-educate joint stability. Techniques used included static kinetic exercises, manual therapy, and active resistance exercises.

For Protocol 2 (Group 2), radial shockwave therapy was applied using the BTL-6000 SWT device. A total of 2000 pulses were delivered per session at a frequency of 10–15 Hz. The intensity was progressively increased from 0.2 to 0.5 BAR depending on patient tolerance. Therapy sessions were conducted twice per week for three weeks, with two days between sessions. Patients were positioned supine, with the affected leg in slight knee flexion and a towel under the popliteal area. Shockwaves were applied to the patellar tendon and the quadriceps insertion. Physical therapy was also performed three times per week, in tandem with shockwave therapy, following the same objectives and techniques as in Protocol 1, the physical therapy program being similar to Group 1.

None of the patients in the two groups received analgesic or anti-inflammatory medication during the study.

### 2.4. Statistical Analysis

The data analysis was performed using SPSS version 29.0.1.0. The Shapiro–Wilk test was used to verify the normality of data distribution, while Levene’s test was employed to assess the homogeneity of variances. Paired *t*-tests were applied for within-group comparisons between initial and final values, while independent *t*-tests were used to compare the mean differences between the two groups. Additionally, Cohen’s d index was calculated to determine the effect size. The effect size according to Cohen’s index was interpreted as small (0.2), medium (0.5), or large (0.8).

Furthermore, Pearson’s correlation coefficient was used to analyse the relationship between the initial and final values of key variables, such as pain (VAS scale), knee flexion range of motion, muscle strength, and suprapatellar perimeter. A cut-off for correlations was considered as follows: values between 0.3 and 0.7 indicated a moderate positive linear relationship, while values between −0.3 and −0.7 indicated a moderate negative linear relationship [30]. This analysis allowed for quantification of the degree of association between pre- and post-intervention measures within each group. For all statistical tests, a *p*-value of less than 0.05 was considered statistically significant.

## 3. Results

Throughout the study, there were no participant dropouts, and all subjects completed the intervention as planned. Furthermore, no adverse effects related to the treatment were reported, demonstrating the safety of both rehabilitation protocols.

The statistical analysis of the paired samples *t*-test, used to compare the initial and final mean values of the VAS for pain in both groups, revealed several key findings. As shown in Table 1, the decrease in VAS scores was highly statistically significant, with a *p*-value of <0.001. This indicates that both rehabilitation protocols were effective in significantly reducing pain levels among the participants.

When comparing the magnitude of pain reduction between the two groups, the difference was greater in Group 2 (shockwave therapy and physical therapy), suggesting that participants in this group experienced a more pronounced reduction in pain compared to Group 1 (low- and medium-frequency electrotherapy, ultrasound, laser therapy, and physical therapy).

The effect size, calculated using Cohen’s d, further supports these findings. As detailed in Table 1, both groups showed large effect sizes, with the reductions in VAS scores being both statistically and clinically significant. The effect size for Group 2 was notably larger, reflecting a greater practical impact of the shockwave therapy combined with physical therapy.

In addition to the *t*-tests, Pearson’s correlation analysis was conducted to assess the relationship between the initial and final VAS scores. For Group 1, there was a very strong positive correlation between the initial and final VAS scores (r = 0.824, *p* < 0.001), indicating a consistent improvement in pain levels following the intervention. Similarly, in Group 2, a strong positive correlation was observed (r = 0.627, *p* < 0.001), further confirming the effectiveness of the shockwave and physical therapy combination in reducing pain.

The analysis of the independent *t*-test did not reveal statistically significant differences between the two groups in terms of initial and final VAS scores. However, as shown in Table 2, the difference in mean values between the groups was reduced in the final assessment, indicating that both rehabilitation programs were effective in significantly lowering pain levels. This suggests that while both interventions led to substantial pain reduction, the magnitude of change between the groups became less pronounced after the application of their respective protocols.

The paired samples *t*-test analysis comparing the initial and final knee flexion degree, goniometrically measured, between healthy and affected knees at the initial and final assessment for the two groups revealed statistically significant improvements (*p* < 0.001), as shown in Table 3. This indicates that both functional rehabilitation protocols positively influenced knee mobility. Specifically, the final knee flexion measurements improved significantly after the interventions, demonstrating enhanced mobility in the affected knee for participants in both groups.

When comparing the magnitude of improvement, the difference between the initial and final knee flexion values was greater in Group 2 (shockwave therapy + physical therapy), suggesting a more pronounced increase in mobility compared to Group 1. The effect size, calculated using Cohen’s d, confirmed a large effect size for both groups, highlighting that the changes in knee flexion were both statistically and clinically significant.

Additionally, Pearson’s correlation analysis for Group 1 showed a strong positive correlation between the initial and final knee flexion values (r = 0.682, *p* < 0.001), indicating consistent improvements. However, no statistically significant correlation was observed in Group 2, despite showing the greater increase in mobility.

The independent *t*-test was employed to compare the two protocols in terms of the initial and final knee flexion differences. As indicated in Table 4, there was a statistically significant difference in the final knee flexion measurements between the two groups (*p* = 0.018). The reduction in the mean difference between the groups in the final evaluation suggests that both rehabilitation programs led to a notable improvement in knee mobility. This indicates that the functional rehabilitation protocols applied in both groups effectively enhanced the mobility of the affected knee.

The paired samples *t*-test analysis comparing the initial and final suprapatellar circumference values between the two groups revealed highly statistically significant differences (*p* < 0.001), as shown in Table 5. These results indicate that the suprapatellar perimeter of the affected knee significantly increased after the implementation of the functional rehabilitation protocols in both groups.

When examining the difference between the initial and final suprapatellar circumference measurements, the increase was more pronounced in Group 2 (shockwave therapy + physical therapy) compared to Group 1 (low- and medium-frequency electrotherapy, ultrasound, laser, and physical therapy), suggesting a greater improvement in muscle mass development in Group 2.

The effect size, calculated using Cohen’s d, was small, indicating that although there were statistically significant differences between the groups regarding suprapatellar circumference, the practical importance of these differences may be limited.

Pearson’s correlation analysis demonstrated a very strong positive correlation between the initial and final suprapatellar circumference values in both groups, with r = 0.998, *p* < 0.001 for Group 1 and r = 0.992, *p* < 0.001 for Group 2. This correlation suggests consistent gains in muscle mass across both protocols.

The enhanced muscle mass in Group 2 may be attributed to the effectiveness of shockwave therapy, which likely facilitated improved proprioception by significantly reducing pain. This allowed the quadriceps to contract more efficiently, enabling a greater increase in muscle volume. Additionally, the ability to apply higher resistance and perform more repetitions without pain in Group 2 contributed to a faster and more effective muscle mass increase compared to Group 1.

The analysis of the independent *t*-test revealed no statistically significant differences between the initial and final values of the suprapatellar perimeter for the two groups (Table 6). However, the reduction in the difference between the means at the final evaluation suggests that both rehabilitation programs effectively increased the suprapatellar perimeter of the affected knee. This indicates that the functional rehabilitation protocols applied in both groups contributed to an increase in muscle mass around the knee.

## 4. Discussion

The primary objective of this study was to assess the efficacy of shockwave therapy combined with specific physiotherapy protocols compared to other traditional approaches, such as low-frequency electrotherapy, laser, ultrasound, and standard physiotherapy programmes. The results indicate that, while both therapeutic protocols were effective in the treatment of patellofemoral syndrome, significant differences were observed in favour of shockwave therapy. This finding suggests that the combination of shockwave therapy and physiotherapy offers additional advantages in terms of pain reduction and functional improvement. This could be attributed to the specific mechanisms of action of shockwave therapy, which promote tissue healing and improve muscle regeneration more effectively than traditional modalities. It is important to note that while conventional treatments remain useful, shockwave therapy could represent a superior therapeutic option for certain patients, particularly those who do not respond adequately to other treatments. Furthermore, the results are consistent with previous studies that have highlighted the potential of shockwave therapy in various musculoskeletal conditions, reinforcing its relevance in clinical practice for optimising outcomes in the management of patellofemoral syndrome [24,25].

Several studies focusing on pain management through transcutaneous electrical nerve stimulation (TENS) have addressed chronic pain. A study conducted in 2000 concluded that TENS could not be recommended for the general management of chronic pain due to the lack of stimulation parameters or concerns regarding its long-term effectiveness. However, a more recent protocol for a literature review on TENS as a pain management tool, published in 2015, concluded that while debate over its use in chronic pain persists, the therapy is effective. Moreover, NICE and Arthritis UK have recommended this therapy as an adjunct to other treatments [31].

In recent decades, extracorporeal shockwave therapy has been widely used in medical practice for managing musculoskeletal disorders, most notably tendinopathies and enthesopathies [32,33,34,35,36]. Due to its effectiveness in providing analgesic effects and in remodelling soft tissue and biostimulation, shockwave therapy has also been successfully used in treating soft tissue injuries following sports injuries and traumatic accidents, such as muscle disorders [37,38], post-traumatic stiff knee [39], and ligament injuries [40,41,42,43]. Additionally, for orthopaedic conditions, shockwave therapy is considered a non-invasive alternative to injections or surgical interventions [44]. Shockwave therapy provides a mechanical stimulus delivered through pulsed acoustic waves, which, via mechanotransduction, are converted into a series of biochemical signals in the targeted tissues, enhancing their regeneration [32,36,45]. This process triggers the production of proteins, nitric oxide, and specific growth factors, initiating a feedback loop that increases neoangiogenesis, tenocyte proliferation, fibroblast activity, and collagen synthesis, thereby improving catabolism, healing, and tissue remodelling [46,47,48,49,50,51].

The acoustic cavitation formed during the negative (traction) phase of the shockwave is another effect of this therapy, as it promotes tissue regeneration by increasing cell membrane permeability and effectively breaking down calcifications in soft tissues [28,31,47]. These biological effects support the use of shockwave therapy for reducing pain, increasing blood flow in ischaemic tissues, softening calcified tissues, treating tissue fibrosis, releasing adhesions, and alleviating post-traumatic knee stiffness, thus improving physical function and sports performance. In terms of pain levels, Table 1 and Table 2 indicate that while both interventions resulted in significant pain reduction, the magnitude of change between the groups became less pronounced after applying their respective protocols. Based on the principle of propagating acoustic energy through biological tissue, shockwave therapy can be divided into two types: focused shockwave therapy and radial shockwave therapy [32,36,52].

The intensity at the focal point of the shockwave, measured as energy flux density (EFD; mJ/mm^2^) per impulse, may influence the therapeutic effects of this type of therapy [53,54].

The differences between the therapeutic effects of focused and radial shockwave therapy have been debated in numerous studies, and each therapy should be considered an independent modality derived from several techniques that generate shockwave impulses [55,56,57,58]. However, it remains unclear whether there is any significant difference between the therapeutic effects of focused and radial shockwaves on musculoskeletal conditions in the lower limbs.

In clinical practice, the energy flux density levels of shockwave therapy range from 0.001 to 0.5 mJ/mm^2^ [52,59,60,61]. Additionally, new studies propose the combined use of focused and radial shockwave therapy [58,59].

Repeated administration of shockwave therapy at very high frequencies may increase the risk of treatment failure [62] and the occurrence of adverse effects [63,64]. Thus, it is crucial to determine the effectiveness of shockwave therapy by identifying the differences in the various intensity levels of its application. The effects of different types of shockwaves, as well as the frequency and intensity of shockwave therapy on musculoskeletal conditions of the lower limbs, should be further investigated, while in the upper limbs studies should compare high- versus low-energy applications [65,66].

Several studies have examined the effectiveness of shockwave therapy in musculoskeletal conditions of the lower limbs, specifically focusing on knee tendinopathy through systematic reviews or meta-analyses [67,68,69]. In addition to patellar tendinopathy, most musculoskeletal conditions of the lower limbs, such as pes anserinus tendinopathy [70], fabella syndrome [71,72], calcification of the popliteus tendon [73], iliotibial band friction syndrome, suprapatellar syndrome [74], and post-traumatic stiffness of tendons and ligaments contributing to joint rigidity [39], have not been included in previous meta-analyses. According to our pilot study, the difference between the initial and final knee flexion values was greater in group 2 (shockwave therapy + physical therapy), indicating a more pronounced increase in mobility compared to group 1 (Table 3 and Table 4). Pain associated with patellofemoral syndrome reduces quadriceps muscle activity, and this reduction in strength is strongly associated with increased symptom severity. The strength and coordination of the thigh, gluteal, and abdominal muscles reduce pressure on the patellofemoral joint by optimising muscle diameter and load distribution across the joint. Therefore, it is recommended to increase muscle strength through pain-free exercises to avoid counterproductive outcomes.

When examining the difference between the initial and final suprapatellar circumference measurements in our study, the increase was more pronounced in group 2 (shockwave therapy + physiotherapy) compared to group 1 (low- and medium-frequency electrotherapy, ultrasound, laser, and physiotherapy), suggesting greater improvement in muscle mass development in group 2. The improved muscle mass in group 2, may be attributed to the efficacy of shockwave therapy, which likely facilitated improved proprioception by significantly reducing pain. This allowed the quadriceps to contract more efficiently, leading to a greater increase in muscle volume. Additionally, the ability to use higher resistance and perform more repetitions without pain in group 2 contributed to faster and more effective muscle mass gain compared to group 1.

Additional interventions that provide short-term pain relief can build patient confidence and improve participation in exercise sessions [75]. A variety of such interventions include manual therapy, foot orthoses, different types of knee joint taping, or applications of cryotherapy and thermotherapy [76,77,78]. Commonly used manual techniques include femoropatellar joint mobilisation, talocrural mobilisation and manipulation (often to improve dorsiflexion), lumbopelvic manipulation, soft tissue mobilisation (often located around the knee joint, on the lateral side), and mobilisation of the femoropatellar and femorotibial joints [76,77,79].

Therapeutic exercise for patients with patellofemoral syndrome is the most evidence-supported management strategy in the specialised literature. Combined hip and knee joint exercises reduce short-term chronic pain and contribute to improved functional capacity in the medium and long term [78]. Resistance exercises to strengthen the hip extensors, abductors, and external rotators [80], quadriceps [81], and core muscles [82] are supported by numerous studies [83,84,85]. More high-quality research is needed to determine the most effective movement parameters to address patellofemoral syndrome, as there is a lack of evidence guiding protocol specificity and exercise dosage [86].

One limitation we faced was the relatively limited number of participants in the study. Another limitation of this study is the lack of long-term follow-up data. Future research should consider extended follow-up periods to better evaluate the sustainability and long-term effects of the intervention. Using computerised functional assessment systems is a future research direction that could help obtain a more accurate interpretation and monitoring of the results obtained by study participants. Another future research direction may involve identifying the effectiveness of shockwave therapy in correlation with participants’ body mass index.

Moreover, with the advancement of artificial intelligence and machine learning methods, future studies should explore this aspect, especially in physical and rehabilitation medicine and tendinopathies, as previously developed studies [87,88].

## 5. Conclusions

This study suggests that the combination of radial shockwave therapy with physiotherapy programmes may offer additional benefits in the treatment of patellofemoral syndrome compared to traditional modalities such as low-intensity electrotherapy, ultrasound, and laser. Patients who received shockwave therapy experienced greater pain reduction, along with more significant improvements in joint mobility and muscle perimeter development. These results could be associated with the specific biological effects of shockwave therapy, including reduced inflammation and enhanced tissue regeneration.

Although the results are promising, they should be interpreted with caution due to the limited sample size and the study’s design. Further research with more robust methodologies is needed to confirm these findings and assess their applicability across different clinical settings. Shockwave therapy, as part of an integrated approach, could be a viable option for certain patients, but its efficacy must be evaluated within the framework of personalised therapeutic strategies.

## Figures and Tables

**Table 1:** Centralization of the data obtained in the paired sample test and Pearson’s correlation for the initial and final mean values of the VAS scale between the two study groups.

	Initial VAS Mean (SD)	Final VAS Mean (SD)	Difference Between Average	Significance Threshold of Paired *t* Test	Cohen Index
Group 1 (protocol 1)	4.93 (±1.13)	1.12 (±0.97)	3.81	*p* < 0.001	5.916
Pearson correlation Index	R = 0.824 *p* ≤ 0.001				
Group 2 (protocol 2)	5.18 (±1.11)	0.71 (±0.81)	4.47	*p* < 0.001	5.082
Pearson correlation Index	R = 0.627 *p* < 0.001				

**Table 2:** Centralization of the data obtained in the independent *t*-test for the initial and final mean values of the VAS scale parameter between the two groups of study participants.

Tested Parameter	Group	Average (±Std. Deviation)	Significance Threshold of Independent *t* Test	Difference Between Average	Cohen Index
Initial VAS	Group 1 (protocol 1)	4.93 (±1.13)	*p* = 0.910	−0.25000	−0.222
	Group 2 (protocol 2)	5.18 (±1.11)			
Final VAS	Group 1 (protocol 1)	1.12 (±0.97)	*p* = 0.360	0.40625	0.453
	Group 2 (protocol 2)	0.71 (±0.81)			

**Table 3:** Centralization of the data obtained in the paired sample test and Pearson correlation for the initial and final mean values of the knee flexion parameter between the two groups of research participants.

	Initial Flexion_Difference Mean (SD)	Final Flexion_Difference Mean (SD)	Difference Between Average	Significance Threshold of Paired *t*-Test	Cohen Index
Group 1 (protocol 1)	1.81 (±1.59)	0.68 (±1.69)	1.13	*p* < 0.001	0.856
Pearson correlation index	R= 0.682 *p* ≤ 0.001				
Group 2 (protocol 2)	3.06 (±1.75)	1.25 (±0.98)	1.81	*p* < 0.001	1.070
Pearson correlation index	R = 0.345 *p* = 0.053				

**Table 4:** Centralization of the data obtained in the independent *t*-test for the initial and final mean values of the knee flexion parameter in the two groups of subjects participating in the study.

Tested Parameter	Group	Average (±Std. Deviation)	Significance Threshold of Independent *t* Test	Difference Between Average	Cohen Index
INITIAL flexion_difference	Group 1 (protocol 1)	1.81 (±1.59)	*p* = 0.428	−1.25000	−0.745
	Group 2 (protocol 2)	3.06 (±1.75)			
FINAL flexion_difference	Group 1 (protocol 1)	0.68 (±1.69)	*p* = 0.018	−0.56250	−0.406
	Group 2 (protocol 2)	1.25 (±0.98)			

**Table 5:** Paired sample test data centralized for initial and final suprapatellar circumference means.

	Initial Perimeter Mean (SD)	Final Perimeter Mean (SD)	Difference Between Average	Significance Threshold of Paired *t*-Test	Cohen Index
Group 1 (protocol 1)	46.48 (±4.52)	47.08 (±4.54)	−0.60	*p* < 0.001	−2.073
Pearson correlation index	R = 0.998 *p* ≤ 0.001				
Group 2 (protocol 2)	46.15 (±5.03)	47.70 (±5.09)	−1.55	*p* < 0.001	−2.396
Pearson correlation index	R = 0.992 *p* < 0.001				

**Table 6:** Centralization of the data obtained in the independent *t*-test for the initial and final mean values of the suprapatellar perimeter between the two groups of study participants.

Tested Parameter	Group	Average (±Std. Deviation)	Significance Threshold of Independent *t*-Test	Difference Between Average	Cohen Index
Initial Perimeter	Group 1 (protocol 1)	46.48 (±4.52)	*p* = 0.321	0.33438	0.070
	Group 2 (protocol 2)	46.15 (±5.03)			
Final Perimeter	Group 1 (protocol 1)	47.08 (±4.54)	*p* = 0.328	−0.62500	−0.129
	Group 2 (protocol 2)	47.70 (±5.09)			

## Data Availability

The data presented in this study may be obtained on request from the corresponding author.

## Supplementary Materials

The following supporting information can be downloaded at: https://www.mdpi.com/article/10.3390/jcm13237260/s1, Supplementary Material S1: A functional rehabilitation programme.
