# Supplementary material for: Contribution of Shockwave Therapy in the Functional Rehabilitation Program of Patients with Patellofemoral Pain Syndrome
Source: J Clin Med. 2024 Nov 29;13(23):7260. doi: 10.3390/jcm13237260 (PMC11641908; doi:10.3390/jcm13237260)
Supplement: Supplementary file 1 [file jcm-13-07260-s001.zip › jcm-3296582-supplementary.pdf]

Supplementary Material S1: Physiotherapy Session Programme

*The following exercise protocol was applied to both groups of subjects in the study.

Exercise 1

Starting Position: Seated on a stationary bike

Action: Perform stationary cycling

Dosage: 10-15 minutes

Observations: None

Exercise 2

Starting Position: Supine on a seat, with hands by the sides

Action: Perform dorsiflexion at the ankle joint and return to the starting position

Dosage: 3 sets of 15 repetitions

Observations: Hold dorsiflexion for 6 seconds on the last repetition of each set

Exercise 3

Starting Position: Standing, knees bent, talocrural joint supported on an elevated surface for stability, with a medium-strength elastic band fixed above the knees

Action: Perform abduction of the lower limbs and return to the starting position

Dosage: 3 sets of 10 repetitions

Observations: None

Exercise 4

Starting Position: Standing with the back against a wall bar and a medium-strength elastic band around the ankles

Action: Flex the hip joint with the knee extended and return to the starting position

Dosage: 3 sets of 10 repetitions for each leg

Observations: None

Exercise 5

Starting Position: Standing with one leg extended forward

Action: Flex the knee joint on one leg and return to the starting position

Dosage: 2 sets of 15 repetitions per leg

Observations: None

Exercise 6

Starting Position: Standing next to a backrest with a medium-strength elastic band around the ankles

Action: Adduct the hip joint with the knee extended and return to the starting position

Dosage: 3 sets of 10 repetitions per leg

Observations: None

Exercise 7

Starting Position: Standing next to a backrest with a medium-strength elastic band around the ankles

Action: Abduct the hip joint with the knee extended and return to the starting position

Dosage: 3 sets of 10 repetitions per leg

Observations: None

Exercise 8

Starting Position: Supine on a mattress, knees bent, feet flat on the floor, arms by the sides, and pelvis tilted

Action: Extend one leg and return to the starting position

Dosage: 2 sets of 10 repetitions per leg

Observations: None

Exercise 9

Starting Position: Prone, with a medium-strength elastic band around the ankles

Action: Flex the calf on the thigh and return to the starting position

Dosage: 2 sets of 10 repetitions per leg

Observations: None

Exercise 10

Starting Position: Standing with bipodal support on a stepper

Action: Perform ankle exercises

Dosage: 10 minutes

Observations: None

Exercise 11

Starting Position: Standing with unipodal support and the knee semi-flexed

Action: Perform a clock pattern with the leg, starting at 12:00 and completing 7 points before returning to the starting position

Dosage: 3 sets of 7 points per leg

Observations: None

Exercise 12

Starting Position: Standing on a balance board with knees bent

Action: Rise onto the toes

Dosage: 2 sets of 15 repetitions

Observations: None

Exercise 13

Starting Position: Sitting on a multifunctional machine

Action: Extend the knee joint against medium resistance

Dosage: 2 sets of 10 repetitions

Observations: None

Exercise 14

Starting Position: Supine on a bench seat, arms by the sides, with the talocrural joint supported by a physioball and knees extended

Action: Perform alternating abduction of the lower limbs

Dosage: 2 sets of 15 repetitions

Observations: None

Exercise 15

Starting Position: Standing with bipodal support on a trampoline and knees bent

Action: Rapidly exchange balls with the therapist to create patient destabilisation

Dosage: 3 sets of 60 seconds

Observations: None

Exercise 16

Starting Position: Standing

Action: Walk backwards on an inclined treadmill at a high incline and low speed

Dosage: 5 minutes

Observations: None

Exercise 17

Starting Position: Standing

Action: Walk forwards on an inclined treadmill at a high incline and medium speed

Dosage: 10 minutes

Observations: None
